# Author Correction: Metabolic reprogramming by Acly inhibition using SB-204990 alters glucoregulation and modulates molecular mechanisms associated with aging

**DOI:** 10.1038/s42003-025-07659-y

**Published:** 2025-02-12

**Authors:** Alejandro Sola-García, María Ángeles Cáliz-Molina, Isabel Espadas, Michael Petr, Concepción Panadero-Morón, Daniel González-Morán, María Eugenia Martín-Vázquez, Álvaro Jesús Narbona-Pérez, Livia López-Noriega, Guillermo Martínez-Corrales, Raúl López-Fernández-Sobrino, Alejandro Castillo-Peña, Lina M. Carmona-Marin, Enrique Martínez-Force, Oscar Yanes, Maria Vinaixa, Daniel López-López, José Carlos Reyes, Joaquín Dopazo, Franz Martín, Benoit R. Gauthier, Morten Scheibye-Knudsen, Vivian Capilla-González, Alejandro Martín-Montalvo

**Affiliations:** 1 Andalusian Molecular Biology and Regenerative Medicine Centre-CABIMER, Universidad de Sevilla-CSIC-Universidad Pablo de Olavide, Seville, 41092 Spain; 2 Center for Healthy Aging, Department of Cellular and Molecular Medicine, University of Copenhagen, Copenhagen, Denmark; 3 Tracked.bio, Copenhagen, Denmark; 4 Instituto de la Grasa (CSIC), Universidad Pablo de Olavide, Sevilla, Spain; 5 Universitat Rovira i Virgili, Department of electronic Engineering & IISPV, Tarragona, Spain; 6 CIBER de Diabetes y Enfermedades Metabólicas asociadas (CIBERDEM), Instituto de Salud Carlos III, Madrid, Spain; 7 Clinical Bioinformatics Area, Fundación Progreso y Salud (FPS), CDCA, Hospital Virgen del Rocio, c/Manuel Siurot s/n, 41013 Sevilla, Spain; 8 Computational Systems Medicine, Institute of Biomedicine of Seville (IBIS), Hospital Virgen del Rocio, Sevilla, 41013 Spain; 9 Bioinformatics in Rare Diseases (BiER), Centro de Investigación Biomédica en Red de Enfermedades Raras (CIBERER), FPS, Hospital Virgen del Rocío, Sevilla, 41013 Spain; 10 FPS/ELIXIR-es, Hospital Virgen del Rocío, Sevilla, 42013 Spain

**Keywords:** Type 2 diabetes, Ageing, Energy metabolism

Correction to: *Communications Biology* 10.1038/s42003-023-04625-4; published online 08 March 2023

In the version of the article initially published, Alejandro Castillo-Peña (Andalusian Molecular Biology and Regenerative Medicine Centre-CABIMER, Universidad de Sevilla-CSIC-Universidad Pablo de Olavide, Seville 41092, Spain) was missing from the author list and has now been added to the HTML and PDF versions of the article.

