# Supplementary material for: Metabolic reprogramming by Acly inhibition using SB-204990 alters glucoregulation and modulates molecular mechanisms associated with aging
Source: Commun Biol. 2023 Mar 8;6:250. doi: 10.1038/s42003-023-04625-4 (PMC9995519; doi:10.1038/s42003-023-04625-4)

Supplementary information

Metabolic reprogramming by Acly inhibition using SB-204990 alters glucoregulation and modulates molecular mechanisms associated with aging

Authors: Alejandro Sola-García[*,1], María Ángeles Cáliz-Molina[*,1], Isabel Espadas[*,1], Michael Petr[2,3], Concepción Panadero-Morón[1], Daniel González-Morán[1], María Eugenia Martín-Vázquez[1], Álvaro Jesús Narbona-Pérez[1], Livia López-Noriega[1], Guillermo Martínez-Corrales[1], Raúl López-Fernández-Sobrino[1], Lina M. Carmona-Marin[2], Enrique Martínez-Force[4], Oscar Yanes[5,6], Maria Vinaixa[5,6], Daniel López-López[7,8,9], José Carlos Reyes[1], Joaquín Dopazo[7,8,9,10], Franz Martín[1,6], Benoit R. Gauthier[1,6], Morten Scheibye-Knudsen[2,3], Vivian Capilla-González[1] and Alejandro Martín-Montalvo[#,1,6].

Affiliations:
[1]Andalusian Molecular Biology and Regenerative Medicine Centre-CABIMER, Universidad de Sevilla-CSIC-Universidad Pablo de Olavide, Seville 41092, Spain
[2]Center for Healthy Aging, Department of Cellular and Molecular Medicine, University of Copenhagen, Copenhagen, Denmark
[3] Tracked.bio, Copenhagen, Denmark
[4]Instituto de la Grasa (CSIC), Universidad Pablo de Olavide, Sevilla, Spain
[5]Universitat Rovira i Virgili, Department of electronic Engineering & IISPV, Tarragona, Spain
[6]CIBER de Diabetes y Enfermedades Metabólicas asociadas (CIBERDEM), Instituto de Salud Carlos III, Madrid, Spain
[7]Clinical Bioinformatics Area, Fundación Progreso y Salud (FPS), CDCA, Hospital Virgen del Rocio, c/Manuel Siurot s/n, 41013, Sevilla, Spain
[8]Computational Systems Medicine, Institute of Biomedicine of Seville (IBIS), Hospital Virgen del Rocio, Sevilla 41013, Spain
[9]Bioinformatics in Rare Diseases (BiER), Centro de Investigación Biomédica en Red de Enfermedades Raras (CIBERER), FPS, Hospital Virgen del Rocío, Sevilla 41013, Spain
[10]FPS/ELIXIR-es, Hospital Virgen del Rocío, Sevilla 42013, Spain
[*]These authors contributed equally
[#]Corresponding author and lead contact

Correspondence: alejandro.martinmontalvo@cabimer.es

Table S1. List of significantly modulated metabolites by GCM in both. HFDSB vs-HFD and STDSB vs-STD experimental groups.

Name	HFDSB-HFD logFC	HFDSB-HFD Pvalue	STDSB-STD logFC	STDSB-STD Pvalue
Pyridine. 2-hydroxy- (1TMS)	-0.753290415	0.029157741	0.696755891	0.045220085
Pyrophosphate (4TMS)	-1.021599248	0.023934549	1.037715039	0.040639926

Table S2. List of significantly altered Pathways using Metaboanalyst 5.0 in STDSS vs. STD comparison. Settings: All pathways; Closeness centrality.

Name	-log10 (p)	Impact (5x)
Pyruvate metabolism	3.4713	2.6642
Cholesterol metabolism	3.2584	0.5991
Oxidative phosphorylation	2.5159	0.61645
Amyotrophic lateral sclerosis (ALS)	2.4485	0.7722
Valine. leucine and isoleucine degradation	2.3807	4.1103
Glutamatergic synapse	2.2596	2.0543
PPAR signaling pathway	2.0318	3.62895
Parkinson´s disease	1.9099	0.4469
Glycolysis or Gluconeogenesis	1.9002	2.1647
Axon guidance	1.8606	0.61465
Glycerolipid metabolism	1.8504	0.71845
beta-Alanine metabolism	1.7848	0.59135
Thermogenesis	1.783	0.62085
Peroxisome	1.7431	0.6154
Dopaminergic synapse	1.7182	1.3677
Arginine and proline metabolism	1.6585	0.9743
Citrate cycle (TCA cycle)	1.6016	6.876
p53 signaling pathway	1.584	4.7604
Hematopoietic cell lineage	1.5056	0.26316
Retinol metabolism	1.482	3.5001
Fatty acid degradation	1.381	0.4559
Cellular senescence	1.3715	1.00615
Retrograde endocannabinoid signaling	1.3696	0.8803
Olfactory transduction	1.3595	0.278685
Propanoate metabolism	1.3427	1.07395
Hippo signaling pathway - multiple species	1.3069	1.3127

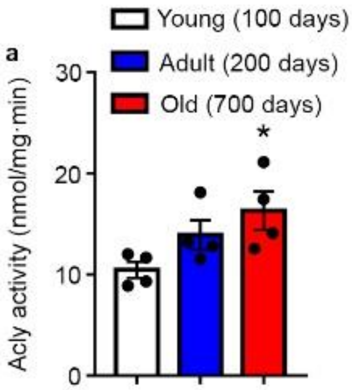

Figure S1, related to Figure 1. Age-dependent increase in hepatic Acly activity.-(a) Hepatic Acly activity. n = 4. One-way ANOVA. Data shown are the means ± SEM. * $p < 0.05$ Old vs. Young.

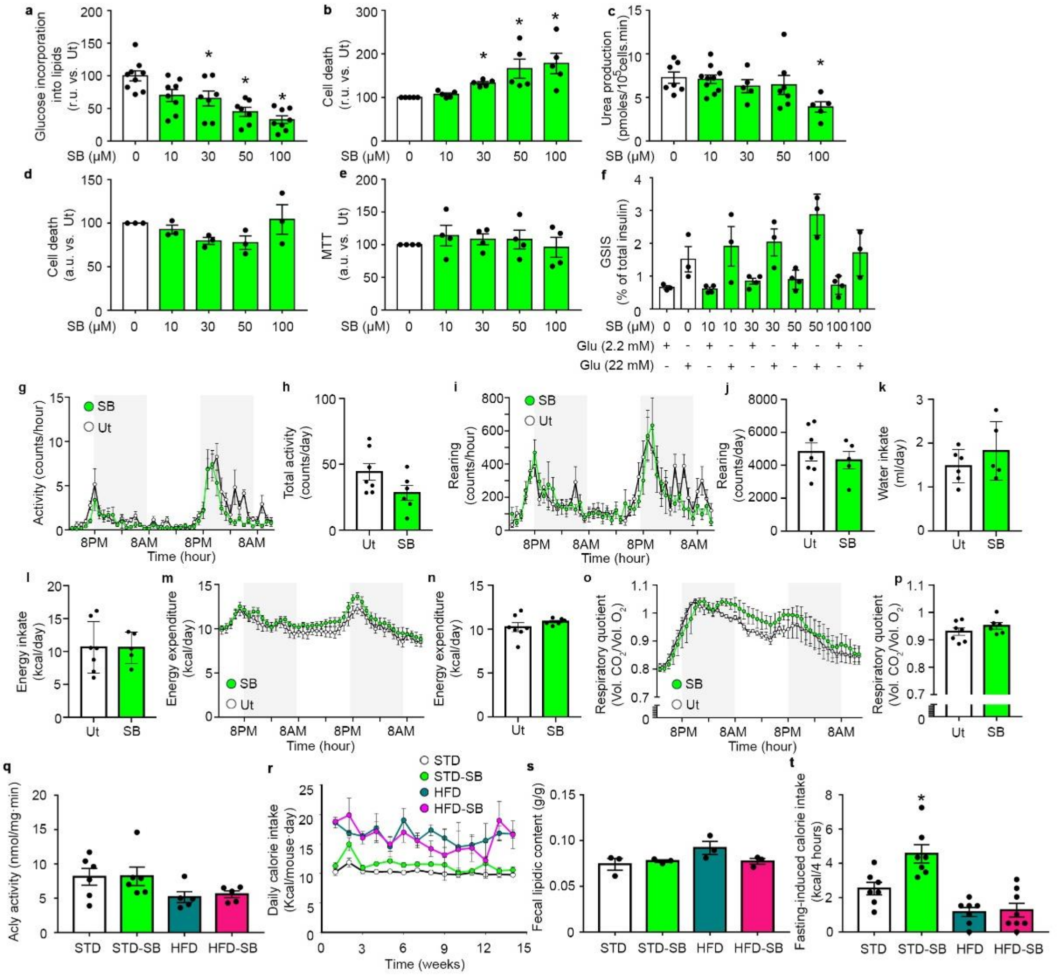

Figure S2, related to figure 2. Effect on viability and functionality of SB-204990 in primary pancreatic islets and hepatocytes. (a-c) Primary hepatocytes were isolated and exposed to different concentrations of SB-204990 for 16 hours. (a) Determination of glucose incorporation into lipids. n = 9 for untreated, n= 8 for 10 µM SB, n = 7 for 30 µM SB, n = 7 for 50 µM SB, n = 8 for 100 µM SB. One-way ANOVA. (b) Cell death analysis by ELISA. n = 5. One-way ANOVA on Ranks. (c) Determination of urea production. n = 7 for untreated, n= 10 for 10 µM SB, n = 5 for 30 µM SB, n = 7 for 50 µM SB, n = 5 for 100 µM SB. One-way ANOVA. (d-f) Primary pancreatic islets were isolated and exposed to different concentrations of SB-204990 for 16 hours. (e) Cell death analysis by ELISA. n = 3. One-way ANOVA on Ranks. (e) Determination of metabolic activity by MTT test. n = 4. One-way ANOVA on Ranks. (f) Determination of glucose stimulated insulin secretion (GSIS). n = 3 for Ut 2.2 mM Glu, Ut 22 mM Glu, 10 µM SB-204990 on 22 mM Glu, 30 µM SB-204990 on 22 mM Glu, 50 µM SB-204990 on 22 mM Glu and 100 µM SB-204990 on 22 mM Glu. n = 4 for 10 µM SB-204990 on 2.2 mM Glu, 30 µM SB-204990 on 2.2 mM Glu, 50 µM SB-204990 on 2.2 mM Glu and 100 µM SB-204990 on 2.2 mM Glu. One-way ANOVA. (g) Hourly spontaneous activity. n = 7 Ut. n = 6 SB. Two-way ANOVA Bonferroni. (h) Daily spontaneous activity. n = 7 Ut. n = 6 SB. Student´s t-test. (i) Hourly rearing activity. n = 7 Ut. n = 5 SB. Two-way ANOVA Bonferroni. (j) Daily rearing activity. n = 7 Ut. n = 5 SB. (k) Water intake during indirect calorimetry. n = 6 Ut. n = 5 SB. Student´s t-test. (l) Daily energy intake during indirect calorimetry. n = 7 Ut. n = 5 SB. Student´s t-test. (m) Hourly energy expenditure. n = 7

Ut. n = 6 SB. Two-way ANOVA Bonferroni. (n) Daily energy expenditure. n = 7 Ut. n = 6 SB. Student´s t-test. (o) Hourly respiratory quotient. n = 7 Ut. n = 6 SB. Two-way ANOVA Bonferroni. (p) Daily respiratory quotient. n = 7 Ut. n = 6 SB. Student´s t-test. (q) Hepatic Acly activity. n = 6 for STD. n = 6 for STD-SB. n = 5 for HFD. n = 5 for HFD-SB. Two-way ANOVA. (r) Average daily energy intake over the course of the study. n = 3. Two-way ANOVA. (s) Fecal lipid content at week 10 of treatment. n = 3. Two-way ANOVA. (t) Fasting-induced energy intake at week 8 of treatment. n = 7 for STD, STD-SB and HFD. n = 8 for HFD-SB. Two-way ANOVA. SB: SB-204990. STD: standard diet. HFD: high fat diet. Ut: untreated. Glu: Glucose. r.u.: relative units. Data shown are the means ± SEM. * $p < 0.05$ SB vs. untreated or STD-SB vs. STD.

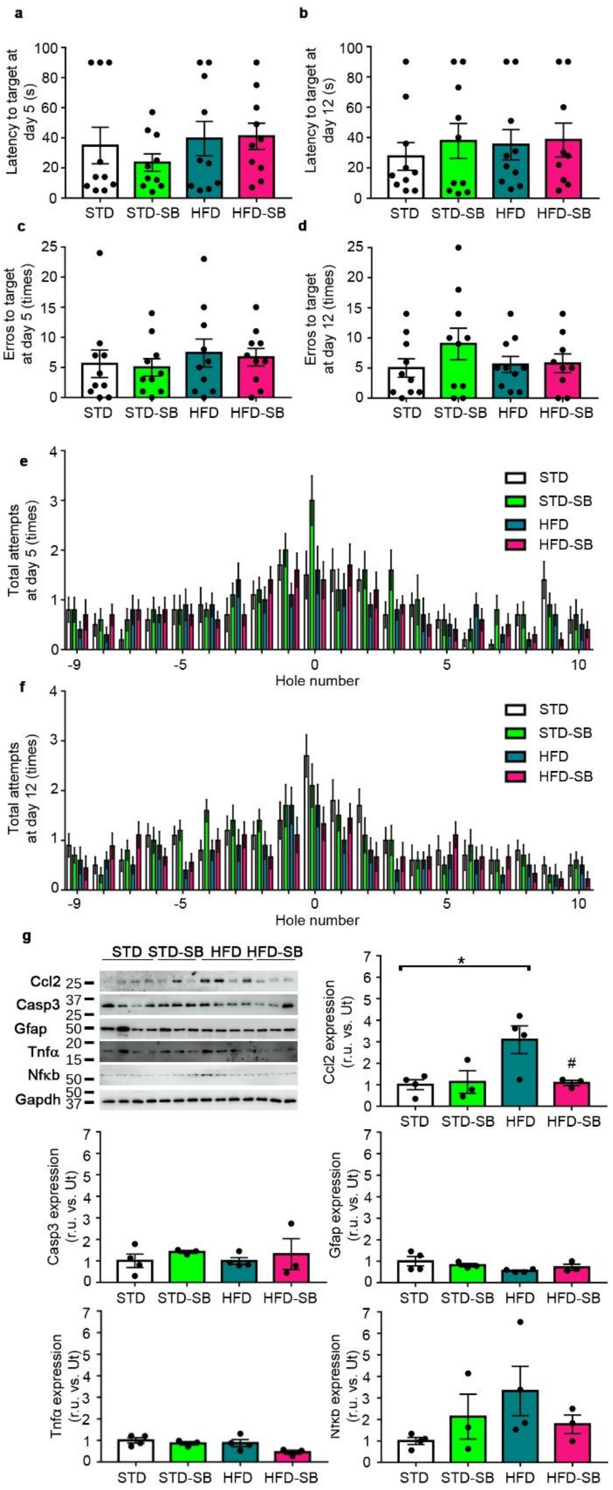

Figure S3, related to figure 3. SB-204990 supplementation does not alter neurocognitive function. (a-f) Spatial memory was assessed by Barnes Maze at week 12-13 of treatment. (a) Latency to target at day 5. n = 10. Two-way ANOVA. (b) Latency to target at day 12. n = 10 for STD. n = 10 for STD-SB. n = 10 for HFD. n = 9 for HFD-SB. Two-way ANOVA. (c) Errors to target at day 5. n = 10. Two-way ANOVA. (d) Errors to target at day 12. n = 10 for STD. n = 10 for STD-SB. n = 10 for HFD. n = 9 for

HFD-SB. Two-way ANOVA. (e) Total attempts to target at day 5. n = 10. Two-way ANOVA. (f) Total attempts to target at day 12. n = 10 for STD. n = 10 for STD-SB. n = 10 for HFD. n = 9 for HFD-SB. Two-way ANOVA. (g) Western blots and densitometry analysis of expression levels of apoptotic and inflammatory markers. n = 4 STD. n = 3 STD-SB. n = 4 HFD. n = 3 HFD-SB. Two-way ANOVA. SB: SB-204990. STD: standard diet. HFD: high fat diet. Data shown are the means ± SEM. * $p < 0.05$ HFD vs. STD or STD-SB vs. STD. # $p < 0.05$ HFD-SB vs. HFD.

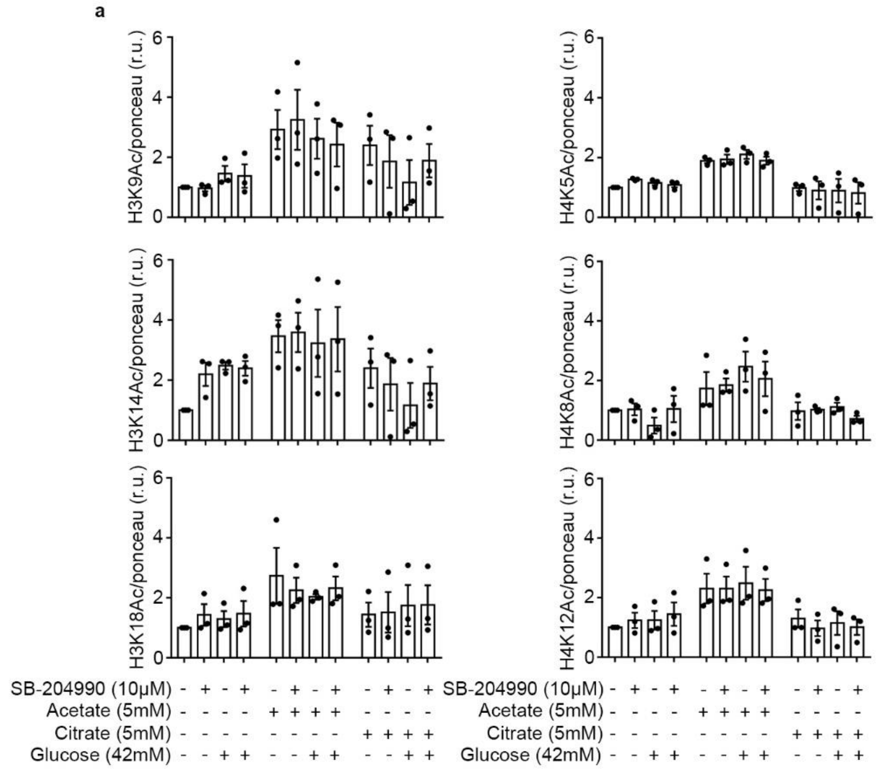

Figure S4, related to figure 4. Quantification of histone acetylation in AML12 cells. AML12 cells were cultured under standard conditions and were treated with indicated doses of acetate, citrate and SB-204990 for 16 hours. Glucose concentration in basal conditions is 17 mM and supplemented is 42 mM. (a) Quantification of histone acetylation levels by western blot of several residues is depicted. n = 3. Two-way ANOVA. SB: SB-204990. r.u.: relative units. Data shown are the means ± SEM. * $p < 0.05$ vs. untreated cells under the same culture conditions. Of note; SB-204990 did not produce significant alterations on histone acetylation.

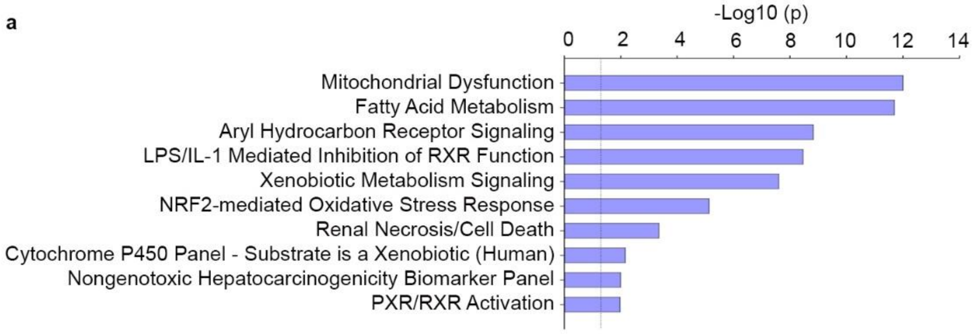

Figure S5, related to figure 5. Multiomic analysis of liver samples. (a) The ToxList module of IPA was used to generate plots depicting the top 10 processes with the lowest p value according to the analytical scheme shown in Figure 5B. Transcriptomic analysis; n = 4 for STD. n = 4 for STD-SB. n = 3 for HFD. n = 3 for HFD-SB. iTRAQ proteomic analysis n = 4. Metabolomic GC/MS analysis; n = 5 for STD. n = 7 for STD-SB. n = 6 for HFD. n = 6 for HFD-SB.

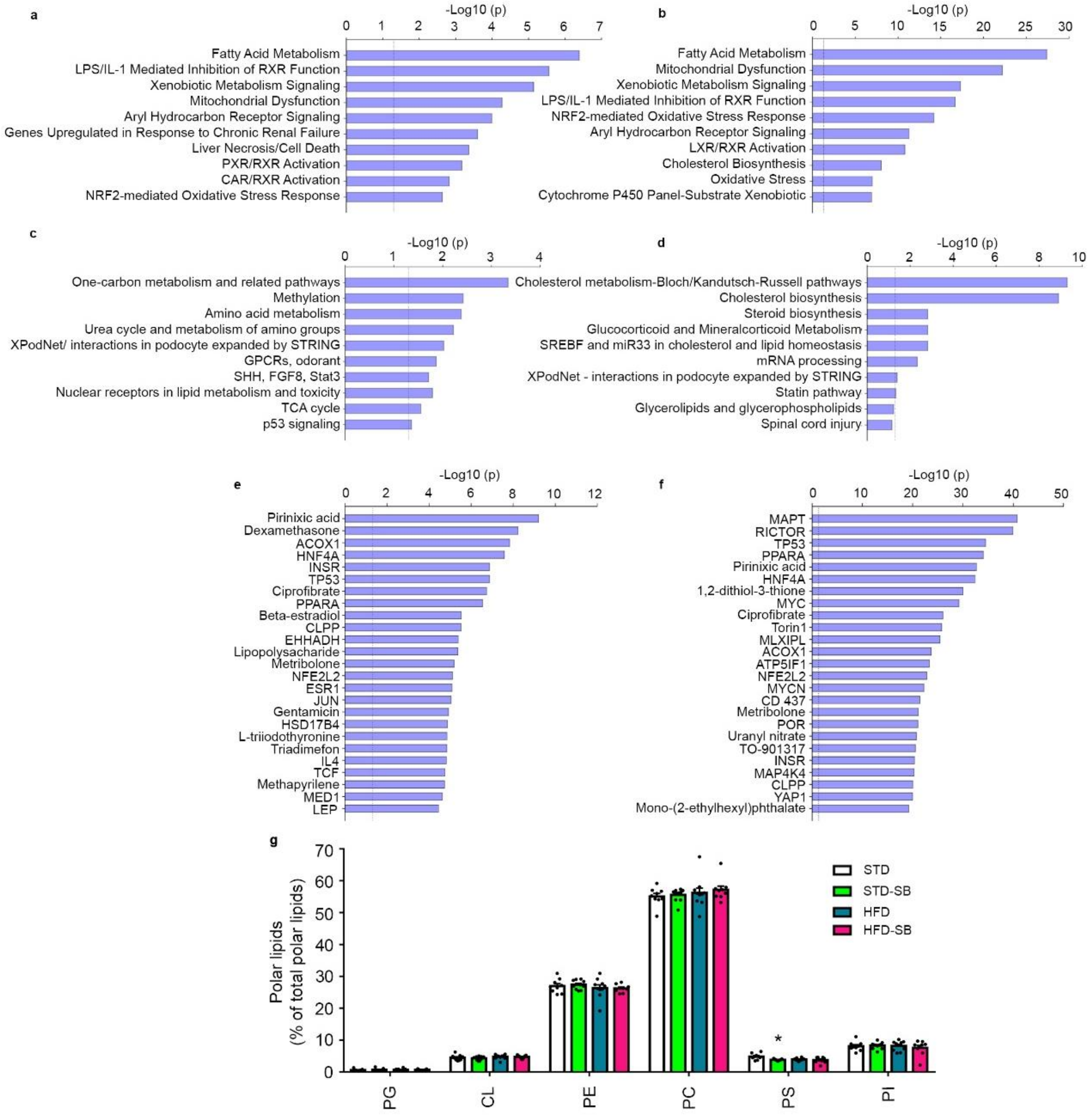

Figure S6, related to figure 6. Hepatic metabolomics in lipid species promoted by SB-204990 in STD-fed and HFD-fed mice. (a-b) Multiomics analysis in liver samples using transcriptomics, proteomics and GC/MS- and LC/MS-based metabolomics. The ToxList module of IPA was used to generate plots depicting the top 10 processes with the lowest p value. (a) STD-SB vs. STD. Transcriptomic analysis; n = 4. iTRAQ proteomic analysis n = 4. Metabolomic GC/MS analysis; n = 5 for STD. n = 7 for STD-SB. Metabolomic LC/MS analysis; n = 6 for STD. n = 7 for STD-SB. (b) HFD-SB vs. HFD. Transcriptomic analysis; n = 3. iTRAQ proteomic analysis n = 4. Metabolomic GC/MS n = 6. Metabolomic LC/MS analysis n = 6. (c-d) The Wikipathways module of the Transcriptional Analysis Console was used using transcriptomic data to generate plots depicting the top 10 pathways with the lowest p value. (c) STD-SB vs. STD. n = 4. (d) HFD-SB vs. HFD. n = 3. (e-f) The Upstream Regulators module of IPA was used to generate plots depicting the top 25 upstream regulators with the lowest p value. (e) STD-SB vs. STD. Transcriptomic analysis; n = 4. iTRAQ proteomic analysis n = 4. Metabolomic GC/MS analysis; n = 5 for STD. n = 7 for STD-SB. Metabolomic LC/MS analysis; n = 6 for STD. n = 7 for STD-SB. (f) HFD-SB vs. HFD. Transcriptomic analysis;

n = 3. iTRAQ proteomic analysis n = 4. Metabolomic GC/MS n = 6. Metabolomic LC/MS analysis n = 6. (g) Hepatic polar lipids. PG: Phosphatidylglycerol. CL: Cardiolipin. PE: Phosphatidylethanolamine. PC: Phosphatidylcholine. PS: Phosphatidylserine. PI: Phosphatidylinositol. n = 9 for STD. n = 11 for STD-SB. n = 10 for HFD. n = 9 for HFD-SB. Two-way ANOVA. SB: SB-204990. STD: standard diet. HFD: high fat diet. Data shown are the means ± SEM. * $p < 0.05$ STD-SB vs. STD. # $p < 0.05$ HFD-SB vs. HFD.

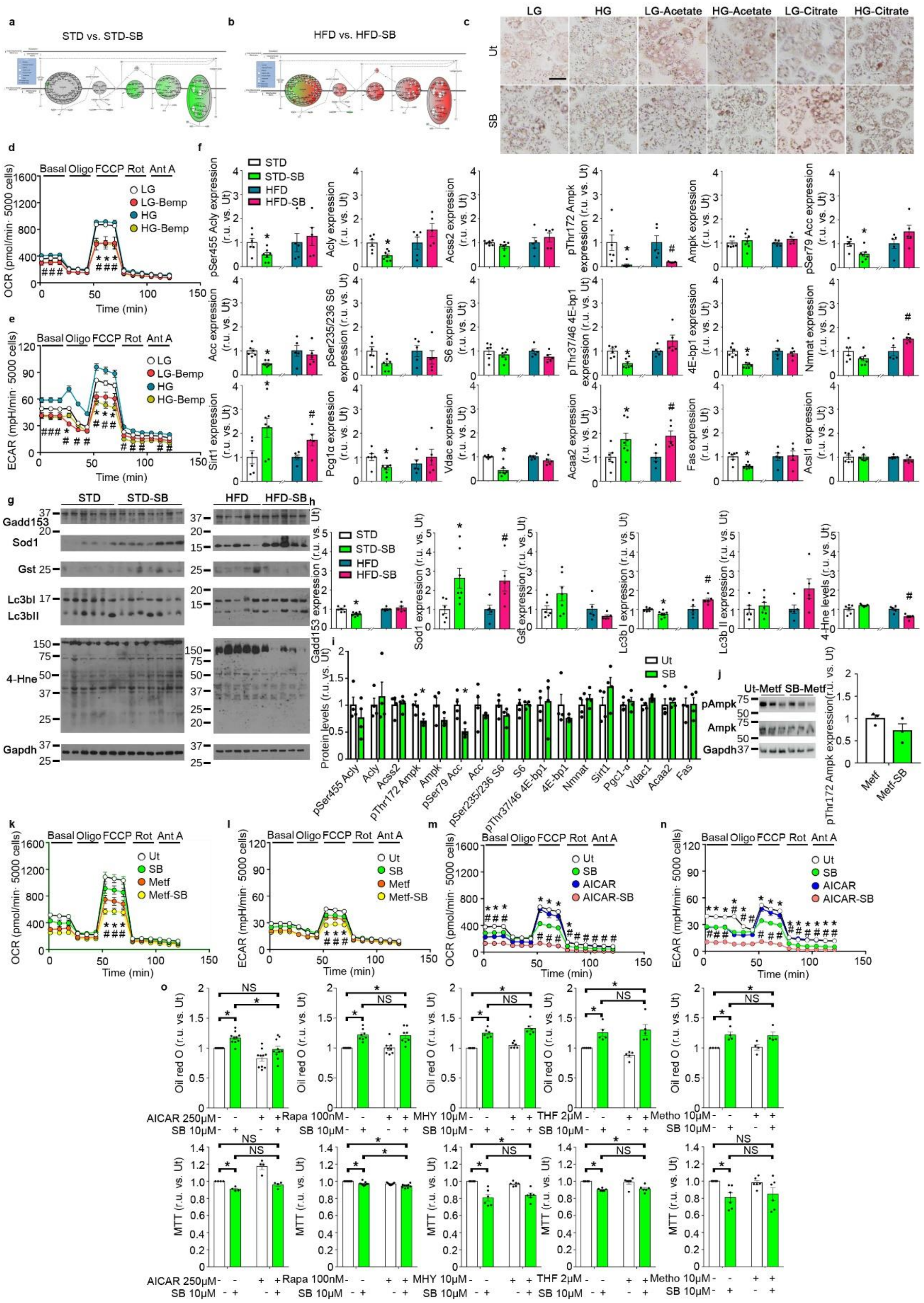

Figure S7, related to figure 7. Effects of SB-204990 in mitochondria and cellular stress. (a-b) Multiomics analysis in liver samples using transcriptomics, proteomics and GC/MS and LC/MS-based metabolomics. Representative picture of the annotated canonical pathway oxidative phosphorylation of IPA. (a) STD-SB vs. STD. Transcriptomic analysis; n = 4. iTRAQ proteomic analysis n = 4. Metabolomic GC/MS

analysis; n = 5 for STD. n = 7 for STD-SB. Metabolomic LC/MS analysis; n = 6 for STD. n = 7 for STD-SB. (b) HFD-SB vs. HFD. Transcriptomic analysis; n = 3. iTRAQ proteomic analysis n = 4. Metabolomic GC/MS n = 6. Metabolomic LC/MS analysis n = 6. (c) Representative images of primary hepatocytes stained with Oil red O treated or not with 10 µM SB-204990 in the presence of acetate or citrate. Scale bar 50 µm. n = 7 (d) Oxygen consumption rate on primary hepatocytes treated with 30 µM bempedoic acid for 16 hours. n = 5 for LG. n = 4 for LG-Bemp. n = 6 for HG. n = 5 for HG-Bemp. Two-way ANOVA. (e) Extracellular acidification rate on primary hepatocytes treated with 30 µM bempedoic acid for 16 hours. n = 5 for LG. n = 4 for LG-Bemp. n = 6 for HG. n = 5 for HG-Bemp. Two-way ANOVA. (f) Densitometric quantification of hepatic proteins by western blot of blots shown in Figure 7K. n = 6 for STD. n = 7 for STD-SB. n = 5 for HFD. n = 5 for HFD-SB. Student´s t-test. (g) Immunoblots for hepatic proteins of mice treated with SB-204990 for 15 weeks. (h) Densitometric quantification of hepatic proteins by western blot. n = 6 for STD. n = 7 for STD-SB. n = 5 for HFD. n = 5 for HFD-SB. Student´s t-test. (i) Densitometric quantification of hepatic proteins by western blot of blots shown in Figure 7L. n = 4. Student´s t-test. (j) Primary hepatocytes were treated with SB-204990 or not in the presence of metformin (500 µM) for 16 hours. Immunoblots for total and phosphorylated Ampk and densitometric quantifications were performed. n = 3 Metformin control. n = 3 Metformin-SB. Student´s t-test. (k) Oxygen consumption rate on primary hepatocytes treated with SB-204990 or not in the presence of metformin (500 µM). n = 5. Two-way ANOVA. (l) Extracellular acidification rate on primary hepatocytes treated with SB-204990 or not in the presence of metformin (500 µM). n = 5. Two-way ANOVA. (m) Oxygen consumption rate on primary hepatocytes treated with SB-204990 or not in the presence of AICAR (250 µM). n = 5. Two-way ANOVA. (n) Extracellular acidification rate on primary hepatocytes treated with SB-204990 or not in the presence of AICAR (250 µM). n = 5. (o) Primary hepatocytes were treated with SB-204990 or not in the presence of AICAR, Rapamycin, MHY1485, Tetrahydrofolic acid or Methotrexate for 16 hours. Quantification of Oil red O staining and metabolic activity by MTT test of primary hepatocytes treated with 10 µM SB-204990 in the presence or not of the indicated compounds. n = 10 in Oil red O test for AICAR experiments. n = 4 in MTT test for AICAR experiments. n = 8 in Oil red O test for Rapamycin experiments. n = 9 in MTT test for Rapamycin experiments. n = 6 in Oil red O test for MHY1485 experiments. n = 6 in MTT test for MHY1485 experiments. n = 5 in Oil red O test for Tetrahydrofolic acid experiments. n = 6 in MTT test for Tetrahydrofolic acid experiments. n = 4 in Oil red O test for Methotrexate experiments. n = 6 in MTT test for Methotrexate experiments. Two-way ANOVA. STD: standard diet. HFD: high fat diet. SB: SB-204990. Bemp: Bempedoic acid. Metf: Metformin. Rapa: Rapamycin. MHY: MHY1485. THF: Tetrahydrofolic acid. Metho: Methotrexate. NS: Not significant. r.u.: relative units. Data shown are the means ± SEM.* $p< 0.05$ SB vs. Ut, LG-Bemp vs. LG, SB-control vs. Ut-control or STD-SB vs. STD. # $p< 0.05$ HG-Bemp vs. HG, SB-Metf vs. Metf, SB-AICAR vs. AICAR or HFD-SB vs. HFD. Other comparisons are highlighted using connector lines and are marked with an asterisk.

Figure S8. Unedited blots shown in the manuscript. Background was set to grey for consistency among panels in main figures.

Blots in figure 4a.

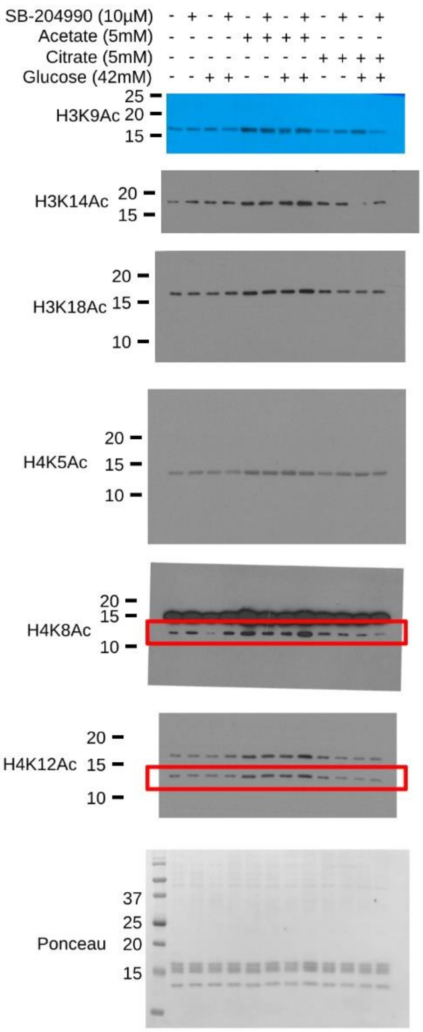

Blots in figure 4b.

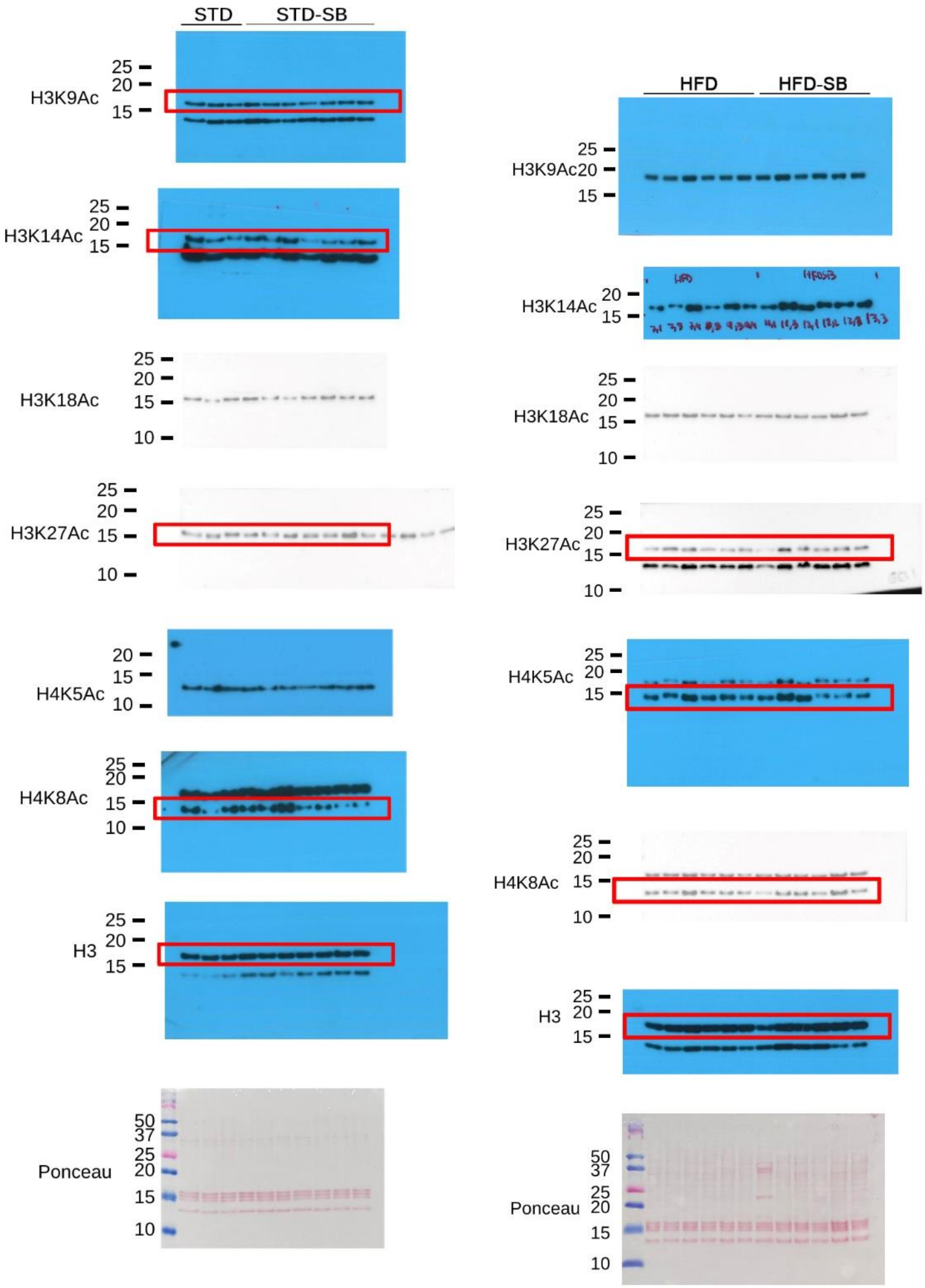

Blots in figure 4d.

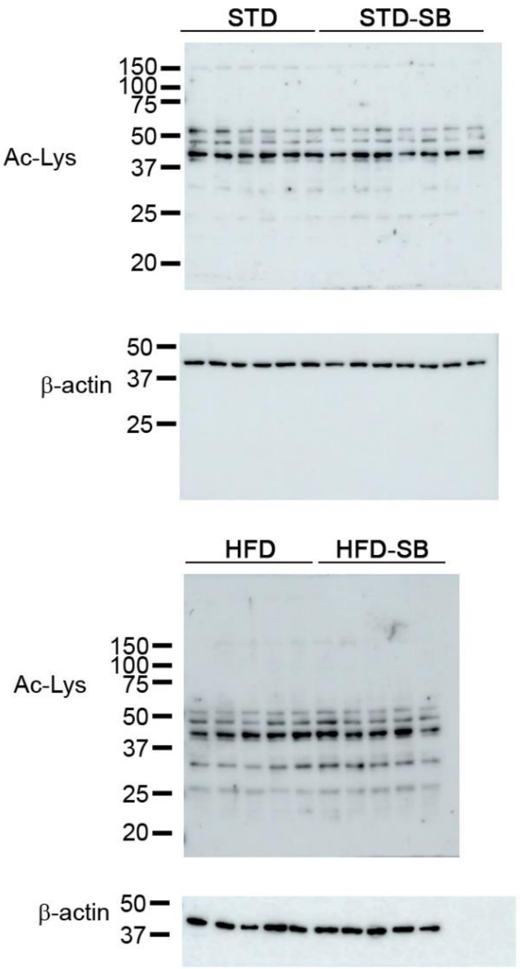

Blots in figure 6e.

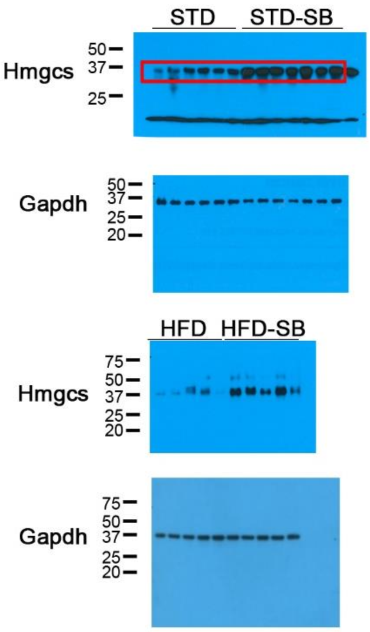

Blots in figure 7 k.

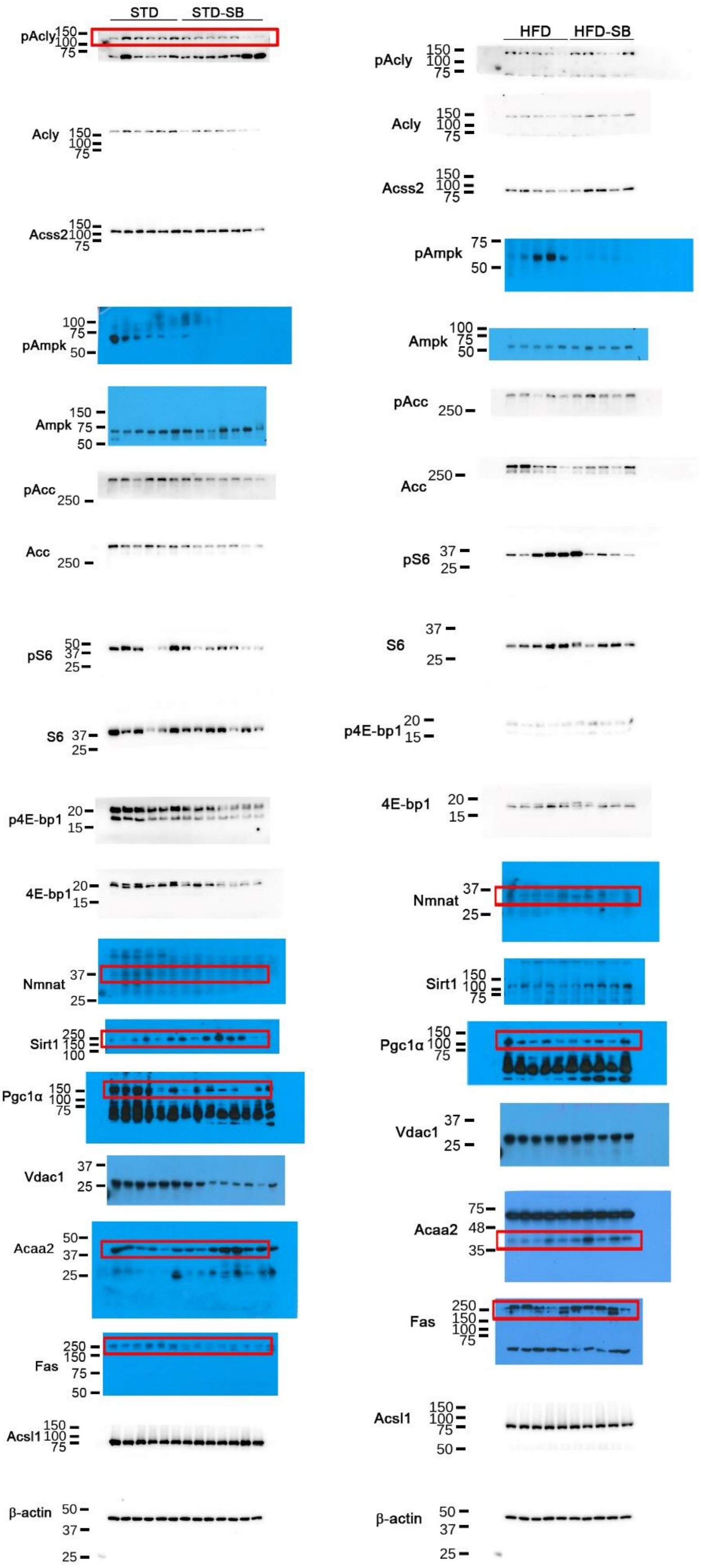

Blots in figure 7l

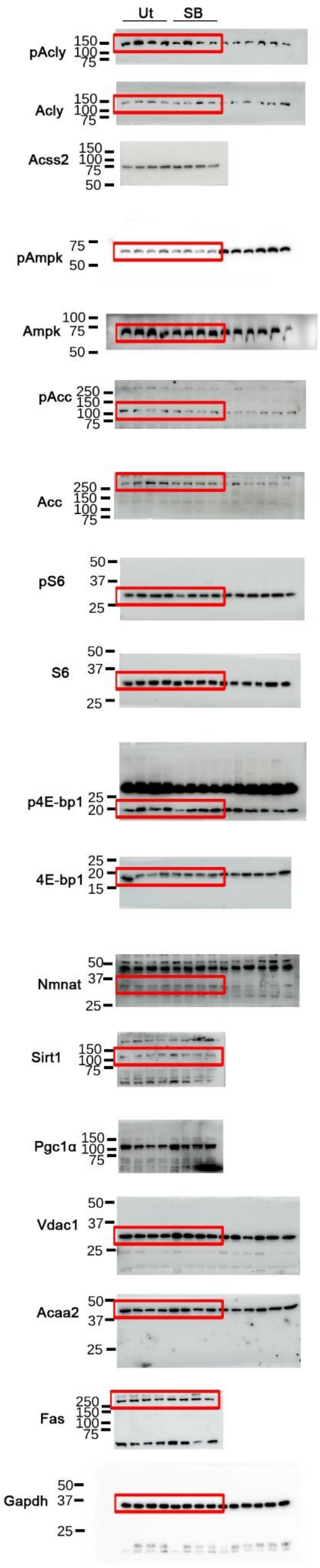

Blots in figure S3g

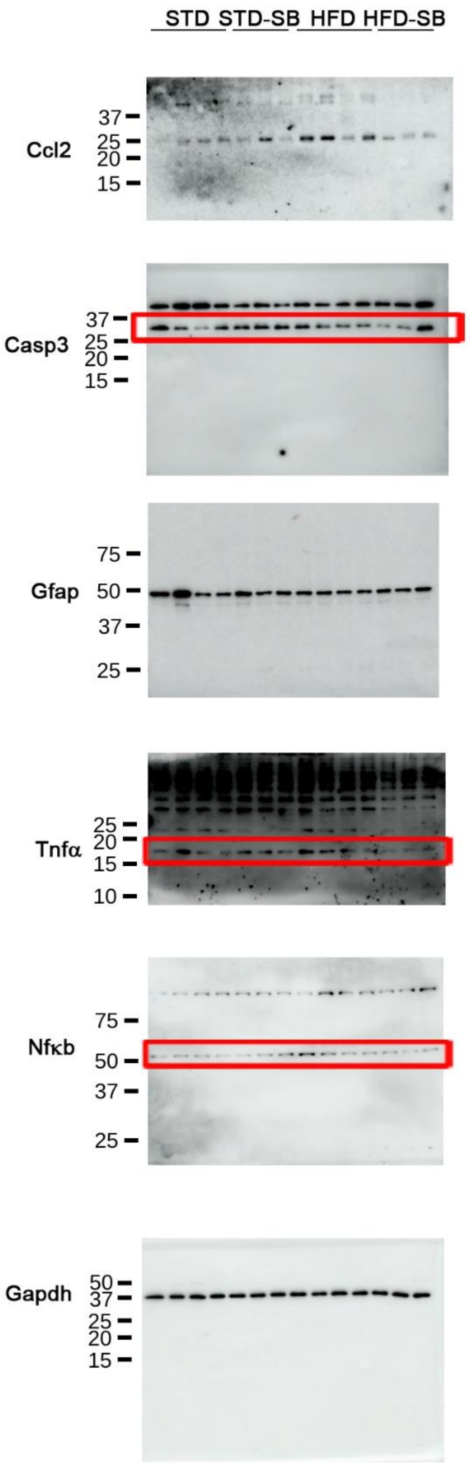

Blots in figure S7g.

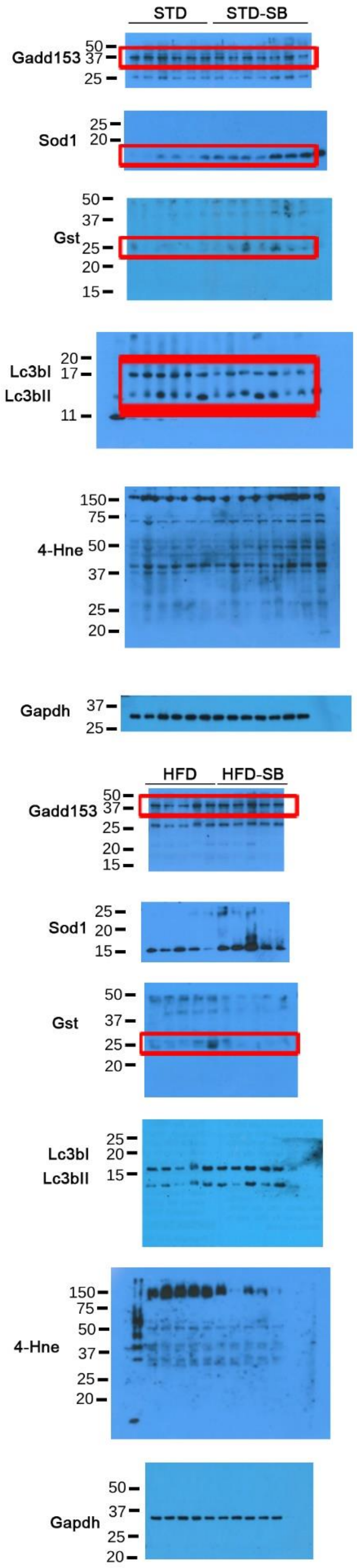

Blots in figure S7j.

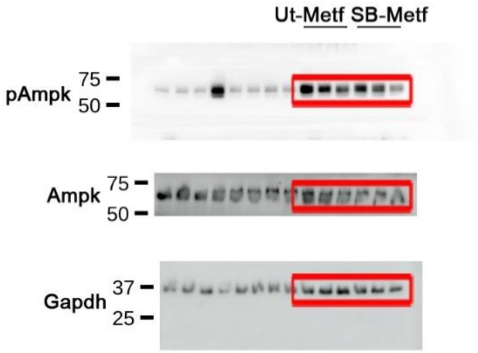

Supplement: Supplementary file 2 — Supplementary Information [file 42003_2023_4625_MOESM2_ESM.pdf]
