# Supplementary material for: Metabolic reprogramming by Acly inhibition using SB-204990 alters glucoregulation and modulates molecular mechanisms associated with aging
Source: Commun Biol. 2023 Mar 8;6:250. doi: 10.1038/s42003-023-04625-4 (PMC9995519; doi:10.1038/s42003-023-04625-4)
Supplement: Supplementary file 3 — Description of Additional Supplementary Files [file 42003_2023_4625_MOESM3_ESM.pdf]

Description of Additional Supplementary Files

File Name: Supplementary Data 1

Description: List of significantly altered transcripts in both, HFDSB vs-HFD and STDSB vs-STD experimental groups.

File Name: Supplementary Data 2

Description: List of significantly altered proteins in both. HFDSB vs-HFD and STDSB vs-STD experimental groups.

File Name: Supplementary Data 3

Description: List of significantly altered Pathways using Metaboanalyst 5.0. Settings: All pathways; Closeness centrality.

File Name: Supplementary Data 4

Description: List of significantly altered Pathways using Metaboanalyst 5.0 in HFDSB vs. HFD comparison. Settings: All pathways; Closeness centrality.

File Name: Supplementary Data 5

Description: List of resources for materials used in this work.
