# Supplementary material for: Metabolic reprogramming by Acly inhibition using SB-204990 alters glucoregulation and modulates molecular mechanisms associated with aging
Source: Commun Biol. 2023 Mar 8;6:250. doi: 10.1038/s42003-023-04625-4 (PMC9995519; doi:10.1038/s42003-023-04625-4)
Supplement: Supplementary file 9 — Reporting Summary [file 42003_2023_4625_MOESM9_ESM.pdf]

nature portfolio

Corresponding author(s): Alejandro Martin-Montalvo

Last updated by author(s): Jan 26, 2023

Reporting Summary

Nature Portfolio wishes to improve the reproducibility of the work that we publish. This form provides structure for consistency and transparency in reporting. For further information on Nature Portfolio policies, see our Editorial Policies and the Editorial Policy Checklist.

Statistics

For all statistical analyses, confirm that the following items are present in the figure legend, table legend, main text, or Methods section.

n/a	Confirmed	
☐	☒	The exact sample size (*n*) for each experimental group/condition, given as a discrete number and unit of measurement
☐	☒	A statement on whether measurements were taken from distinct samples or whether the same sample was measured repeatedly
☐	☒	The statistical test(s) used AND whether they are one- or two-sided<br>*Only common tests should be described solely by name; describe more complex techniques in the Methods section.*
☒	☐	A description of all covariates tested
☐	☒	A description of any assumptions or corrections, such as tests of normality and adjustment for multiple comparisons
☐	☒	A full description of the statistical parameters including central tendency (e.g. means) or other basic estimates (e.g. regression coefficient) AND variation (e.g. standard deviation) or associated estimates of uncertainty (e.g. confidence intervals)
☒	☐	For null hypothesis testing, the test statistic (e.g. *F*, *t*, *r*) with confidence intervals, effect sizes, degrees of freedom and *P* value noted<br>*Give P values as exact values whenever suitable.*
☒	☐	For Bayesian analysis, information on the choice of priors and Markov chain Monte Carlo settings
☒	☐	For hierarchical and complex designs, identification of the appropriate level for tests and full reporting of outcomes
☒	☐	Estimates of effect sizes (e.g. Cohen's *d*, Pearson's *r*), indicating how they were calculated

Our web collection on statistics for biologists contains articles on many of the points above.

Software and code

Policy information about availability of computer code

Data collection	It was not required
Data analysis	It was not required

For manuscripts utilizing custom algorithms or software that are central to the research but not yet described in published literature, software must be made available to editors and reviewers. We strongly encourage code deposition in a community repository (e.g. GitHub). See the Nature Portfolio guidelines for submitting code & software for further information.

Data

Policy information about availability of data

All manuscripts must include a data availability statement. This statement should provide the following information, where applicable:

- Accession codes, unique identifiers, or web links for publicly available datasets
- A description of any restrictions on data availability
- For clinical datasets or third party data, please ensure that the statement adheres to our policy

Data supporting the results of the study will be made available from the corresponding author upon reasonable request.
Trancriptomic data: Gene Expression Omnibus GSE196853
Metabolomic data: Zenodo DOI: 10.5281/zenodo.6222260.

Proteomic data: Zenodo DOI: 10.5281/zenodo.6140992.

Human research participants

Policy information about studies involving human research participants and Sex and Gender in Research.

Reporting on sex and gender	It was not required.
Population characteristics	It was not required.
Recruitment	It was not required.
Ethics oversight	It was not required.

Note that full information on the approval of the study protocol must also be provided in the manuscript.

Field-specific reporting

Please select the one below that is the best fit for your research. If you are not sure, read the appropriate sections before making your selection.

☒ Life sciences ☐ Behavioural & social sciences ☐ Ecological, evolutionary & environmental sciences

For a reference copy of the document with all sections, see nature.com/documents/nr-reporting-summary-flat.pdf

Life sciences study design

All studies must disclose on these points even when the disclosure is negative.

Sample size	Sample size was based on previous exprience of the researchers. No formal power calculations were perfomed.
Data exclusions	Data was not excluded from the analyses except in the case of technical issues during experimentation resulting in low quality results not meeting quality criteria. In metabolomic analyses one sample was excluded due to degradation.
Replication	The n in each experiments is shown in figure legends. Each n represents a biological replicate. Only biological replicates are shown in this study, and no technical ones.
Randomization	Animals were randomized according to the starting body weight.
Blinding	In vivo research was performed blind when possible due to major differences of the phenotype (for instance, differences in appearance in HFD-induced obese mice and experiments at different ages in mice are quite obvious). In vitro experiments were not performed blinded.

Reporting for specific materials, systems and methods

We require information from authors about some types of materials, experimental systems and methods used in many studies. Here, indicate whether each material, system or method listed is relevant to your study. If you are not sure if a list item applies to your research, read the appropriate section before selecting a response.

Materials & experimental systems

n/a	Involved in the study	
☐	☒	Antibodies
☐	☒	Eukaryotic cell lines
☒	☐	Palaeontology and archaeology
☐	☒	Animals and other organisms
☒	☐	Clinical data
☒	☐	Dual use research of concern

Methods

n/a	Involved in the study	
☒	☐	ChIP-seq
☒	☐	Flow cytometry
☒	☐	MRI-based neuroimaging

Antibodies

Antibodies used	Histone-H3 Cell Signaling Technology 4499T<br>H3K9ac Cell Signaling Technology 9649T<br>H3K14ac Cell Signaling Technology 7627T

	H3K18ac Cell Signaling Technology 13998T<br>H3K27ac Cell Signaling Technology 8173T<br>H4K5ac Cell Signaling Technology 8647T<br>H4K8ac Cell Signaling Technology 2594T<br>H4K12ac Cell Signaling Technology 13944T<br>Acetylated lysine Cell Signaling Technology 9441<br>Anti-Rabbit IgG HRP-linked Cell Signaling Technology 7074P2<br>Hmgcs Santa Cruz Biotechnology SC-166763<br>Gapdh Cell Signaling Technology Cat# 2118, RRID:AB_561053<br>pSer455 Acly Cell Signaling Technology 4331<br>Acly Cell Signaling Technology 4332<br>AceCS1 Cell Signaling Technology 3658<br>Nmnat Santa Cruz Biotechnology Sc-98249<br>Sirt1 Millipore 07-131<br>pThr172 Ampk Cell Signaling Technology Cat# 2535, RRID:AB_331250<br>Ampk Cell Signaling Technology Cat# 2532, RRID:AB_330331<br>pSer79 ACC Cell Signaling Technology 11818<br>ACC Cell Signaling Technology 3676<br>Pgc1a Santa Cruz Biotechnology Cat# sc-517380, RRID:AB_2755043<br>Vdac1 Abcam Cat# ab15895, RRID:AB_2214787<br>Acaa2 Santa Cruz Biotechnology Cat# sc-100847, RRID:AB_2219392<br>Fas Santa Cruz Biotechnology Cat# sc-55580, RRID:AB_2231427<br>Acsl1 Cell Signaling Technology 9189<br>pThr37/46 4E-bp1 Cell Signaling Technology 2855<br>4E-bp1 Cell Signaling Technology 9452<br>pSer 235/236 S6 Cell Signaling Technology 4856<br>S6 Cell Signaling Technology 2217<br>β-actin Sigma-Aldrich A5441<br>Gadd153 Santa Cruz Biotechnology Cat# sc-575, RRID:AB_631365<br>Sod1 Abcam Cat# ab13498, RRID:AB_300402<br>Gst Sigma-Aldrich Cat# G1160, RRID:AB_259845<br>Lc3b Cell Signaling Technology Cat# 2775, RRID:AB_915950<br>4-hne Millipore Cat# 393206-100UL, RRID:AB_211975<br>Anti-mouse IgG HRP Sigma-Aldrich A-9044<br>Anti-rabbit IgG HRP Sigma-Aldrich A-0545<br>Ccl2 Enzo Life Sciences Cat# ALX-804-465-C100, RRID: AB_2050978 1:200<br>Casp3 Cell Signaling Technology Cat# 9662, RRID: AB_331439 1:1000<br>Gfap Sigma-Aldrich Cat# G3893, RRID: AB_477010 1:1000<br>Tnfα Proteintech Cat# 26405-1-AP 1:1000<br>NfκB Abcam Cat# ab32536, RRID: AB_776751 1:10000<br>Anti-mouse HRP-IgGκ light chain Santa Cruz Biotechnology Cat# sc-516102, RRID:AB_2687626
Validation	All antibodies used were purchased from well-stablished companies (Cell Signaling Technology, Santa Cruz Biotecnology n Sigam.-Aldrich, Millipore, ABCAM...). All antibodies are validated according to manufacterer's product description as shown on their website.

Eukaryotic cell lines

Policy information about cell lines and Sex and Gender in Research

Cell line source(s)	AML-12 cells were purchased from ATCC.
Authentication	AML-12 cells were authenticated based on their distinctive hepatocyte morphology.
Mycoplasma contamination	Cells were tested negative for micoplasma contamination.
Commonly misidentified lines (See ICLAC register)	These cell lines were not used.

Animals and other research organisms

Policy information about studies involving animals; ARRIVE guidelines recommended for reporting animal research, and Sex and Gender in Research

Laboratory animals	Mus musculus: C57BL/6
Wild animals	Wild animals were not used.
Reporting on sex	Experiments were performed in male mice.
Field-collected samples	Field-collected samples were not used.
Ethics oversight	Experimentation was approved by the CABIMER Animal Committee and the Junta de Andalucía.

Note that full information on the approval of the study protocol must also be provided in the manuscript.
